# Leptin Deficiency, Caused by Malnutrition, Makes You Susceptible to SARS-CoV-2 Infection but Could Offer Protection from Severe COVID-19

**DOI:** 10.1128/mSphere.00031-21

**Published:** 2021-05-12

**Authors:** Dewald Schoeman, Burtram C. Fielding

**Affiliations:** a Molecular Biology and Virology Research Laboratory, Department of Medical Biosciences, University of the Western Cape, Bellville, Cape Town, Western Cape, South Africa; University of Maryland School of Medicine

**Keywords:** COVID-19, SARS-CoV-2, undernutrition, leptin deficiency, cytokine storm, malnutrition

## Abstract

In much of the developing world, severe malnutrition is the most prevalent cause of immunodeficiency and affects up to 50% of the population in some impoverished communities. As yet, we do not know how severe acute respiratory syndrome coronavirus 2 (SARS-CoV-2) will behave in populations with immunodeficiency caused by malnourishment. Interestingly, researchers are now speculating that, in some instances, a defective cellular immune system could paradoxically be a protective factor against severe disease in certain patients contracting SARS-CoV and SARS-CoV-2. This could be linked to the absence of T-cell activation. Based on available information presented here, it is plausible that the hyperimmune response, and subsequent cytokine storm often associated with severe coronavirus disease 2019 (COVID-19), could be “counteracted” by the defective immune response seen in individuals with malnutrition-induced leptin deficiency. In this paper, we proposed a theory that although those with malnutrition-linked leptin deficiency are at risk of SARS-CoV-2 infection, they are at lower risk of developing severe COVID-19.

## Abstract (precis)

In much of the developing world, severe malnutrition is the most prevalent cause of immunodeficiency and affects up to 50% of the population in some impoverished communities. As yet, we do not know how severe acute respiratory syndrome coronavirus 2 (SARS-CoV-2) will behave in populations with immunodeficiency caused by malnourishment.

## OPINION/HYPOTHESIS

In most cases, infection with the four common human coronaviruses (HCoVs) causes mild cold-like symptoms, involving the upper respiratory tract. A severe lower respiratory tract infection (LRTI) can, however, develop in immunocompromised patients. Unlike 229E, OC43, NL63, and HKU1, the more pathogenic severe acute respiratory syndrome coronavirus (SARS-CoV) and Middle East respiratory syndrome coronavirus (MERS-CoV) cause LTRI, accompanied by a cytokine storm in patients with poor prognosis (1).

A novel coronavirus (severe acute respiratory syndrome coronavirus 2 [SARS-CoV-2], or 2019-nCoV) has been shown to cause acute respiratory disease syndrome (ARDS) in humans. The typical clinical symptoms of coronavirus disease 2019 (COVID-19) include fever, dry cough, shortness of breath, and fatigue. A small percentage of patients, however, develop various fatal complications, including septic shock, organ failure, pulmonary edema, severe pneumonia, and ARDS (2). As of 1 December 2020, COVID-19 has been confirmed in 61.8 million people in more than 200 countries and territories, resulting in 1.4 million deaths worldwide, a case mortality of approximately 2.3% (3). Now, accumulating evidence suggests that a majority of SARS-CoV-2-positive patients with severe COVID-19 might also develop a cytokine storm syndrome (4). In particular, the elderly and people with preexisting medical conditions appear more susceptible to severe COVID-19 outcome, which may be associated with a cytokine storm and ARDS (5). There is no consensus definition of “cytokine storm” that encompasses all the various therapies, pathogens, cancers, autoimmune conditions, and monogenic disorders that can trigger such an event. Nevertheless, it can generally be regarded as an excessive immune response, one that may be greater than its immediate benefit, that causes collateral damage to organs, and is accompanied by elevated levels of circulating cytokines (6). Although the exact cause of the cytokine storm in severe COVID-19 is not yet clear, it stands to reason that the SARS-CoV-2 virus plays a central role, since similar features have been observed in SARS-CoV, MERS-CoV, as well as other viral infections (7). This paper will focus on the immunosuppressive effect of leptin deficiency-linked malnutrition in the context of SARS-CoV-2, and the concomitant hyperinflammatory response seen in severe COVID-19 (6).

Viral infections can trigger secondary/acquired hemophagocytic lymphohistiocytosis (sHLH)—a hyperinflammatory syndrome—mainly resulting in an unremitting fever, cytopenias, and pulmonary involvement (including ARDS) in about half of patients (8). Left untreated, sHLH leads to a cytokine storm and hemophagocytosis, resulting in fatal hypercytokinemia with multiorgan failure (9). In severe lung infections, the cytokine storm-linked inflammatory response can move into the systemic circulation, producing systemic sepsis, which could result in the damage of other organs (10). Interestingly, severe COVID-19 is associated with a cytokine profile similar to that of sHLH (11), and increasing evidence shows elevated levels of inflammatory cells, cytokines, chemokines, and other inflammatory mediators associated with severe COVID-19 (1, 12–14). Since COVID-19 also closely resembles SARS, and ARDS progression in severe SARS is marked by a similar elevation in inflammatory cytokines, it is reasonable to conclude that similar immunopathogenic features are to be expected in severe COVID-19 cases (15). Although ARDS and the accompanying respiratory failure are the leading causes of death in severe COVID-19 cases, the hyperinflammatory response from the cytokine storm facilitates the immunopathology that culminates in ARDS and respiratory failure (16). In fact, inflammation from the hyperactivated immune response increases capillary permeability and damages the alveolar-capillary membrane, bringing about pulmonary edema, impairing gas exchange, and often culminating in respiratory failure, characteristic features of ARDS (1, 17).

**FIG 1:**
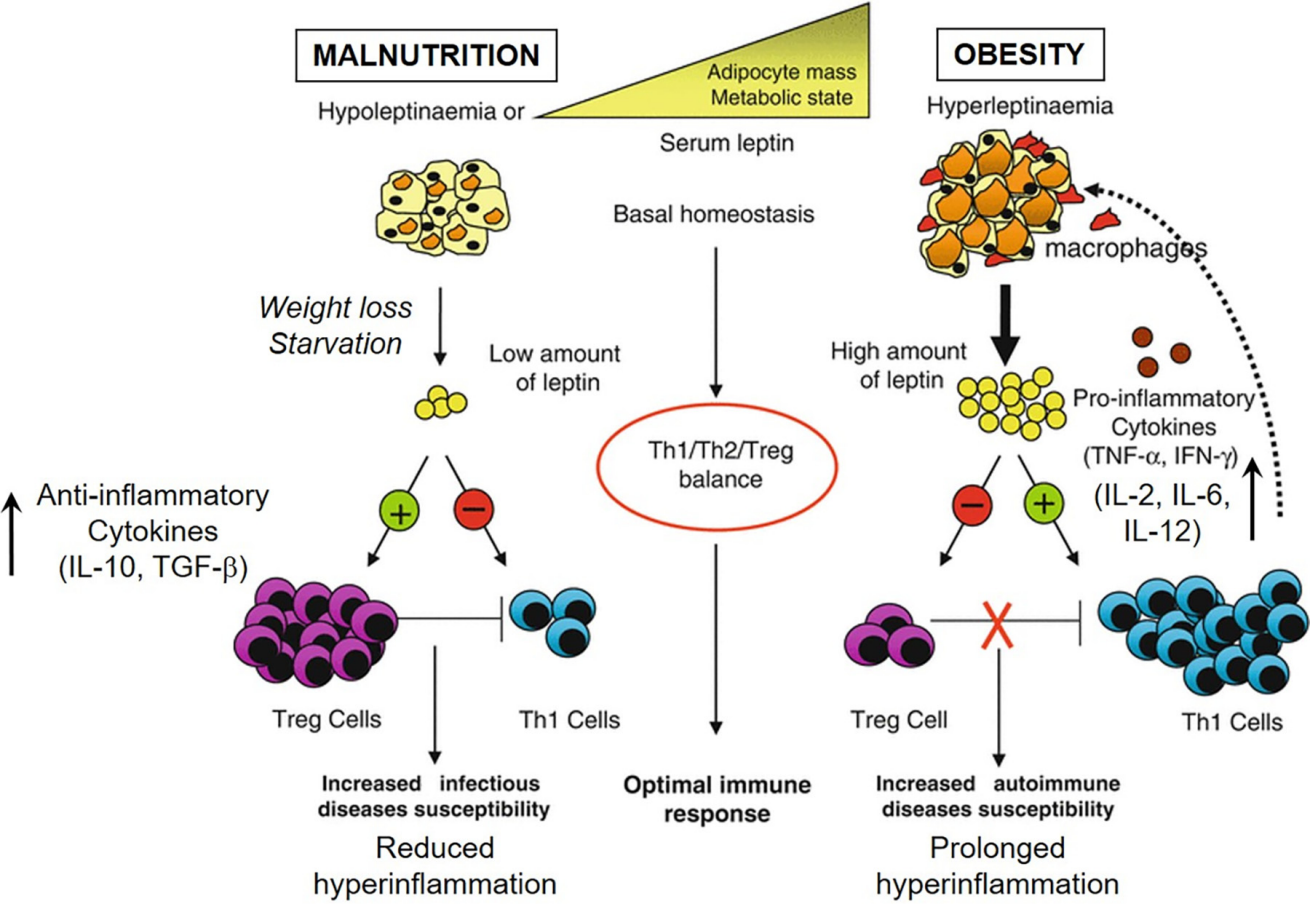
Leptin modulates the immune response by acting on both effector T (Teff) cells and regulatory T (Treg) cells. Under homeostatic conditions, adipocytes secrete basal levels of leptin that positively regulate Th1 immunity through the proliferation of Teff cells and, simultaneously, regulate Treg cell expansion negatively. This achieves an optimal immune profile—a balance between Teff and Treg cells, whereby Tregs mediate immune tolerance through anti-inflammatory cytokine production, while Th1 cells confer protection from infections through proinflammatory cytokine production. Reduced leptin levels (hypoleptinaemia), as a consequence of weight loss or malnutrition (starvation), reduce Teff cell proliferation and increase Treg cell expansion. This downregulates Th1 immunity and produces an immune response skewed toward an anti-inflammatory profile, increasing a person’s susceptibility to infections by reducing the Th1-mediated inflammatory response. Accordingly, malnourished persons are predisposed to contracting infections but can be protected from hyperinflammatory conditions by this anti-inflammatory profile. Conversely, high leptin secretion (hyperleptinaemia), attributed to increased adipocyte mass as seen in obesity, triggers Teff expansion and reduces Treg cell proliferation. This skews the immune response toward a proinflammatory profile, increasing the person’s risk to hyperinflammatory conditions by reducing the Treg-mediated anti-inflammatory response. Accordingly, such persons are predisposed to hyperinflammatory conditions and can experience more severe inflammatory responses on account of the proinflammatory immune profile. Adapted from reference 40.

Malnutrition, and protein energy malnutrition (PEM) in particular, has been linked to an immunosuppressive phenotype. This phenotype is characterized by reduced proinflammatory cytokines, notably interleukin 2 (IL-2), IL-6, IL-12, gamma interferon (IFN-γ), tumor necrosis factor alpha (TNF-α); an impaired response from inflammatory T-cells (CD69^+^, CD25^+^), dendritic cells (DCs) (HLA-DR^+^), and macrophages (CD14^+^) upon stimulation; and a concomitant increase in anti-inflammatory cytokines, such as IL-4, IL-10, and transforming growth factor beta (TGF-β) (18–20). These studies demonstrate that PEM brings about an immunological shift in the balance of cytokines, favoring an anti-inflammatory response, and this is in stark contrast to what has been reported for a cytokine storm; a hyperinflammatory event in which proinflammatory cytokine levels are increased and those of anti-inflammatory cytokines are decreased (Fig. 1). Clearly, the cytokine profile of persons suffering from PEM is inverse to that of a cytokine storm, as many of the damaging, proinflammatory cytokines from the storm are inversely regulated to anti-inflammatory cytokines. This immunosuppressive phenotype, however, gives rise to a phenomenon called “immunoparalysis” —a persistently high anti-inflammatory immune response following infection. Since a proper immunological balance between pro- and anti-inflammatory processes is vital for recovery from critical illness, it has been proposed that patients who survive the cytokine storm, but subsequently die, may be those who did not recover from immunoparalysis (21). Accordingly, malnutrition does not necessarily protect against COVID-19 itself but rather appears to counteract the severe complications brought on by the cytokine storm as a consequence of the infection.

Malnourished individuals commonly have decreased levels of the adipokine, *viz*. leptin, and this deficiency in leptin has been linked to the impaired immune function typically seen in malnourished persons (22–24). Leptin has been shown to shift the immune response from an anti-inflammatory profile to a more proinflammatory one, validating the observed immunosuppressive phenotype seen in such leptin-deficient malnourished persons (25, 26). This effect has been demonstrated in animal models and other infectious diseases, where subsequent administration of leptin also reversed the effects of leptin deficiency observed on immunosuppression (6, 27). This specifically demonstrated that severe protein-restricted (malnourished) BALB/c mice, infected with visceral leishmaniasis, expressed significantly higher levels of anti-inflammatory cytokines (IL-4, IL-10, and TGF-β) compared to infected mice on a regular protein diet. Subsequent administration of leptin to these malnourished, infected mice significantly decreased the expression of IL-4, IL-10, and TGF-β, directly implicating leptin in shifting the immune response from an anti-inflammatory profile to a proinflammatory one. Human clinical studies on malnutrition-linked immunodeficiency would, therefore, benefit greatly from measuring the leptin levels of malnourished persons and comparing it to that of well-nourished persons to determine whether such a significant difference could support the observed differences in their immunological profile. One study measured the serum leptin levels in both SARS-CoV-2-infected and uninfected patients and reported a positive correlation between the serum leptin levels and body mass index (BMI) of infected persons (28). Although the authors did not include immunological markers, they described a clinical and biological framework in which they proposed that the increased BMI and accompanying serum leptin levels could explain the need for SARS-CoV-2-infected patients to receive mechanical ventilation, implicating the increased BMI and serum leptin levels in acute pulmonary inflammation.

Several studies have used BMI as the only marker for obesity. However, we caution against this. The use of BMI alone as a marker for obesity is an accepted Western norm, which we recommend should be accompanied by additional adiposity markers such as waist circumference, waist-to-hip/height ratio, and/or body fat percentage to classify persons more accurately as overweight or obese. Accordingly, S. A. Peters et al. (29) reported that higher adiposity markers, *viz.* BMI, waist circumference, waist‐to‐hip ratio, and waist‐to‐height ratio, are associated with a greater risk of mortality from COVID-19. T. Yates et al. (30) also reported a dose-response association between BMI and waist circumference and testing positive for COVID-19, and C. Razieh et al. (31) similarly reported a difference in the risk of COVID-19 between ethnic groups based on BMI and waist circumference. Furthermore, J. Wang et al. (32) used both BMI and serum leptin levels, reporting that patients with a high BMI have significantly higher levels of leptin. This is also positively associated with inflammatory mediators and disease severity in such patients.

Admittedly, we cannot generalize, and our hypothesis might not be applicable to all developing countries due to innate differences in diet and nutritional states, among other factors. Also, our hypothesis might not be relevant to all elderly persons in developing countries since “immunosenescence” and “inflammaging” could also contribute to COVID-19 age-related mortality. A marked decrease in both CD4^+^ helper T cells—that help to clear viral infections—and immunosuppressive T cells (Tregs) (“immunosenescence”), accompanied by a chronic, low-grade inflammatory state from increased serum inflammatory mediators (“inflammaging”) would be additional factors relevant for consideration in this elderly population (33).

### Implications of leptin deficiency-induced immunosuppression for biomarkers and vaccine development.

Worryingly, the data from these studies suggest that malnutrition could also cause a deficient vaccine response. The importance of both DCs and IL-2 in the development of a functional immunological memory strongly suggests that a reduced vaccine response could likely be the result of impaired DC function, insufficient IL-2 levels to prime effector memory T cells, and an impaired response from effector memory T cells that have formed (34, 35). This could also have dire implications for vaccine administration as immunodeficiency-linked malnutrition might not simply be “corrected” by vaccination. These studies clearly implicate a metabolic requirement in the mounting and maintenance of functional immunological memory, which is paramount to an effective vaccine response (25, 26).

A neglected aspect of the leptin-immune axis that could also have implications for vaccine development, is the effect of leptin on antibody production. Leptin downregulates antibody production and class switching, and since serum leptin levels are elevated in both obese and elderly persons, this should be considered to ensure the development of effective vaccines for these vulnerable groups (36). Interestingly, this study also found that rapamycin rescues the negative leptin-induced effect on B cells. Furthermore, the impact of leptin on the immune system and its correlation with disease progression and severity would make it a valuable predictive tool, or biomarker, for predicting patient prognosis (32). Naturally, its efficacy as a biomarker would first have to be tested and confirmed, but it could prove especially useful in Africa and other developing countries, where treatments are often sparse and expensive and not everyone has access to primary health care. Leptin’s use as a biomarker could also assist in developing a risk-based approach where a patient’s leptin levels could be used to evaluate whether the patient could benefit from early, prophylactic treatment, such as dexamethasone or any other relevant COVID-approved treatment(s).

In this paper, we proposed that individuals with leptin deficiency-linked immunosuppression due to malnutrition are not at higher risk of developing severe COVID-19 compared to healthy, well-nourished individuals with physiologically “normal” leptin levels. Malnourished persons can still contract COVID-19, but they are less likely to die from the hyperinflammation brought on by the cytokine storm. Interestingly, researchers have now speculated that a defective cellular immune system—as seen in HIV patients—“could paradoxically be a protective factor in some patients” contracting SARS-CoV (37). Similarly, for SARS-CoV-2, “the compromised immunity might be the reason that HIV/AIDS patients did not occur inflammatory changes and clinical symptoms” associated with severe COVID-19 (38), which could be linked to the absence of T-cell activation (39). Based on available information, it is plausible that the hyperimmune response, and subsequent cytokine storm often associated with severe COVID-19, could be “counteracted” by the defective immune response seen in individuals with malnutrition-induced leptin deficiency.
